# Heavy reliance on plants for Romanian cave bears evidenced by amino acid nitrogen isotope analysis

**DOI:** 10.1038/s41598-020-62990-0

**Published:** 2020-04-20

**Authors:** Yuichi I. Naito, Ioana N. Meleg, Marius Robu, Marius Vlaicu, Dorothée G. Drucker, Christoph Wißing, Michael Hofreiter, Axel Barlow, Hervé Bocherens

**Affiliations:** 1 Department of Geosciences, Biogeology, University of Tübingen, Hölderlinstraße 12, 72074 Tübingen, Germany; 2 Nagoya University Museum, Nagoya University, Furo-cho, Chikusa-ku, Nagoya, 464-8601 Japan; 3 “Emil Racoviță” Institute of Speleology, Romanian Academy, Calea 13 Septembrie, nr. 13, 050711 Sector 5, Bucharest, Romania; 4 Senckenberg Centre for Human Evolution and Palaeoenvironment (S-HEP), University of Tübingen, Hölderlinstraße 12, 72074 Tübingen, Germany; 5 Institute for Biochemistry and Biology, Faculty for Mathematics and Natural Sciences, University of Potsdam, Karl-Liebknecht-Str. 24-25, 14476 Potsdam, OT Golm Germany; 6 School of Science and Technology, Nottingham Trent University, Clifton Lane, Nottingham, NG11 8NS UK

**Keywords:** Palaeontology, Biogeochemistry, Palaeoecology

## Abstract

Heavy reliance on plants is rare in Carnivora and mostly limited to relatively small species in subtropical settings. The feeding behaviors of extinct cave bears living during Pleistocene cold periods at middle latitudes have been intensely studied using various approaches including isotopic analyses of fossil collagen. In contrast to cave bears from all other regions in Europe, some individuals from Romania show exceptionally high *δ*^15^N values that might be indicative of meat consumption. Herbivory on plants with high *δ*^15^N values cannot be ruled out based on this method, however. Here we apply an approach using the *δ*^15^N values of individual amino acids from collagen that offsets the baseline *δ*^15^N variation among environments. The analysis yielded strong signals of reliance on plants for Romanian cave bears based on the *δ*^15^N values of glutamate and phenylalanine. These results could suggest that the high variability in bulk collagen *δ*^15^N values observed among cave bears in Romania reflects niche partitioning but in a general trophic context of herbivory.

## Introduction

Bears represent the largest terrestrial members within the Carnivora alive today and the vast majority of them have carnivorous or omnivorous feeding habits. Until around 25,000 years ago, the coldest period in the Pleistocene, additional, now extinct bear species were living^1–4^, among which the so-called cave bears, a very large type of bear that formed the sister lineage of extant brown bears and polar bears (e.g., ref. ^5^). The paradox of the cave bear is that their diet has been said to be herbivorous despite their large body sizes while extant herbivorous Carnivora species are smaller^6,7^. After their divergence from the brown bear lineage 1.2–1.6 million years ago, cave bear populations showed substantial morphological and genetic variability and multiple forms have been recognized^8^, although their taxonomic status and the relationships among them continue to be debated^9^. The possible causes of the extinction of these bears are also intensively debated, involving climate change, human impacts, and (lack of) flexibility in feeding behavior^10–15^. Understanding cave bear feeding behavior is therefore important as it might give insights into the extinction of this species, and also it could be relevant for the conservation of extant, herbivorous carnivoran species that are under threat of extinction (e.g., binturong, red panda, giant panda^16,17^).

More recent studies have shown mixed results based on different lines of evidence including anatomical properties like craniodental morphologies, tooth wear analyses, mortality patterns (e.g., sex ratio), etc. wherein the conclusions were highly context dependent and differed by sample-sets^18–23^. This is also the case for stable carbon and nitrogen isotope analyses (*δ*^13^C and *δ*^15^N) on collagen extracted from bone/teeth^24–31^. Relatively low *δ*^15^N values of most of these bears so far indicate their highly-plant-dependent feeding habits with possible exceptions for some groups in today’s Romania that exhibited relatively high *δ*^15^N values^30,32,33^. The *δ*^15^N value of bulk bone/teeth collagen is known to increase from prey to its consumer in a food web, thus being an important indicator to evaluate the trophic position of organisms^34,35^. Other factors also influence *δ*^15^N values of collagen, such as variability in the consumed plants and environmental factors including climate^36–38^, leading to possible spatiotemporal shifts of the isotopic baseline due to heterogeneities among local environments^39–42^. Hence, some herbivores may exhibit similar *δ*^15^N values as predatory species in the same context^30,43–45^.

**Figure 1:**
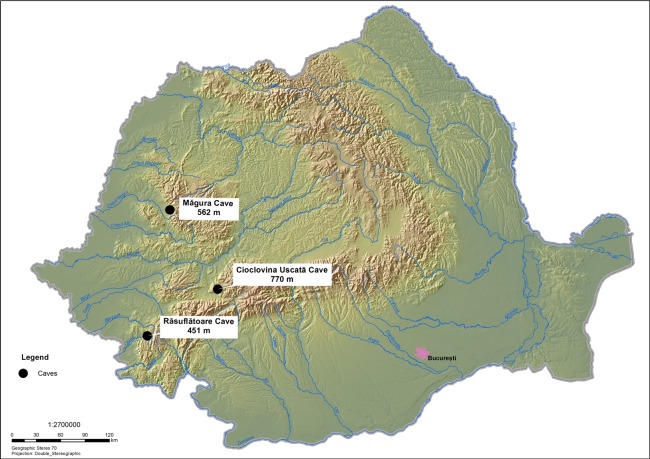
Map showing locations of the cave sites in Romania mentioned in the text.

To solve this uncertainty, a new isotopic approach has been developed. Indeed, it has been demonstrated that *δ*^15^N values of individual amino acids (AA) can be classified into several categories, “trophic” and “source” AAs that exhibit more ^15^N enrichment and less/little ^15^N enrichment, respectively, in each trophic step of food webs^46–53^. It is worth mentioning that the comparison of the “source” AAs like phenylalanine (Phe) with the “trophic” AAs like glutamate (Glx) enables the evaluation of animal trophic position regardless of baseline *δ*^15^N fluctuations in ecosystems^54^. We aim at testing the two hypotheses that high collagen *δ*^15^N values observed for cave bears in the Romanian region were attributed to (i) omnivorous/carnivorous feeding behavior associated with a trophic level effect as a corollary or (ii) consumption of plants with high bulk *δ*^15^N values. To this end, *δ*^15^N values of individual AAs in collagen were measured for adult cave bears from several sites in this region (Fig. 1). One cave lion and one horse have been analyzed together with the adult cave bear collagen to represent end-members for trophic positions for a terrestrial carnivore and a terrestrial herbivore in the Late Pleistocene.

## Results

**Figure 2:**
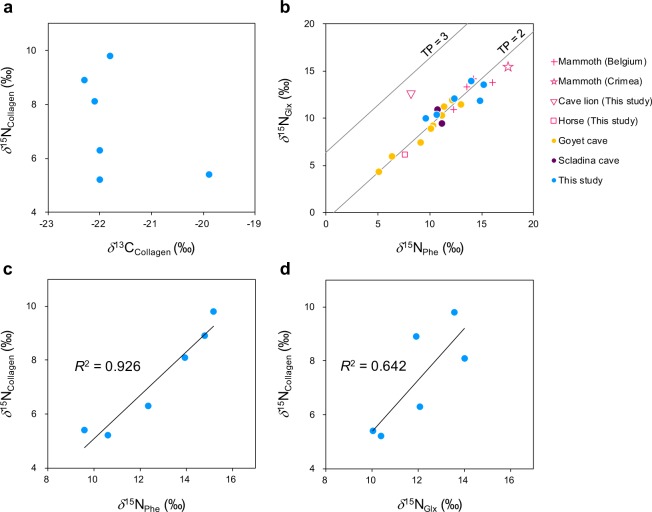
Isotope results for the cave bears investigated from the three cave sites in Romania. Scatterplots of: (**a**) *δ*^13^C and *δ*^15^N values of bulk collagen; (**b**) *δ*^15^N values of glutamate and phenylalanine. The solid lines indicate the theoretical lines for TP = 2 and TP = 3, respectively, in terrestrial C_3_-vascular-plant ecosystems. The spacing between these lines indicate the inter trophic enrichment (see Methods). Values for mammoth and cave bears from Belgium were taken from the literature^57,58,60^; (**c**) *δ*^15^N values of bulk bone collagen and phenylalanine; (**d**) *δ*^15^N values of bulk collagen and glutamate.

Judging from the C content (26.0–46.1%), N content (9.1–16.2%) and C/N atomic ratio (3.3–3.5), all the cave bear as well as the cave lion and horse bone collagen are well preserved (Table 1)^55,56^. Large variations in *δ*^13^C and *δ*^15^N values of bulk collagen for the cave bears were observed: −19.9 to −22.3‰ for *δ*^13^C and 5.2 to 9.8‰ for *δ*^15^N values, respectively (Fig. 2). The bears sampled from each cave corresponded to different time horizons. Out of six cave bear samples, two have been molecular dated (USR10 from Măgura cave and USR67 from Răsuflătoarei cave), and the other four have been radiocarbon dated (Table 1). Based on calibrated ^14^C age and the mean of the posterior samples for molecular dated samples, ages of the samples from Măgura fell within the interval 28–31 ka BP, while the oldest bears were from Cioclovina Uscată with ^14^C ages of c. 43 and 48 cal ka BP. The samples from Răsuflătoarei cover a wider time-span (20–49 ka BP), due to the wide confidence interval depicted based on molecular tip dating, given that USR67 is the only representative within its clade. In each cave and related timespan, both high (above 8‰) and lower *δ*^15^N values of bulk collagen have been recorded (see more details in Table 1).

**Table 1:** Collagen samples for cave bears from the three Romanian caves and reference fauna analyzed in this study.

Lab. ID	Common name/Species name	Site	Uncalibrated ^14^C date/Molecular tip date^*^ (yrs BP)	Calibrated dates (95.4%) (cal BC)/95% credibility interval for molecular estimates*	Lab. number	*d*^13^C (‰)	*d*^15^N (‰)	%C	%N	C/N atom
USR10	Cave bear/−	Măgura	30,866*	28,885-32,518*	—	−22.0	6.3	26.0	9.1	3.3
USR11	Cave bear/−	Măgura	24,615 ± 101	28,392-28,891	ETH-87394	−22.1	8.1	36.8	12.3	3.5
USR22	Cave bear/−	Cioclovina Uscată	40,567 ± 594	43,125-45,230	ETH-87395	−22.0	5.2	33.9	11.8	3.3
USR25	Cave bear/−	Cioclovina Uscată	43,119 ± 807	44,990-48,401	ETH-87396	−22.3	8.9	36.7	12.9	3.3
USR65	Cave bear/−	Răsuflătoarei	31,005 ± 183	34,541-35,333	ETH-95736	−21.8	9.8	38.3	13.5	3.3
USR67	Cave bear/−	Răsuflătoarei	38,313*	19,932-49,692*	—	−19.9	5.4	46.1	16.2	3.3
*Reference fauna*										
LOM 16	Horse /*Equus ferus*	Lommersum				−20.9	4.2	38.8	13.7	3.3
Leo 01	Cave lion /*Panthera spelaea*	North Sea	37110 ± 1070			−19.5	8.3	36.8	13.1	3.3

Strong correlations between the *δ*^15^N values of several AAs and those of bulk collagen were observed for the investigated cave bears, among which phenylalanine showed the strongest one (*R*^2^ = 0.926, *P* < 0.01). Other AA such as hydroxyproline (*R*^2^ = 0.704, *P* < 0.05) also showed strong correlations with bulk collagen *δ*^15^N values. The scatterplot presenting *δ*^15^N_Phe_ *vs*. *δ*^15^N_Glx_ values shows that some cave bear individuals from the Romanian caves exhibited isotope values similar to those analyzed in previous studies for cave bears from Late Pleistocene Belgian sites, though three individuals with bulk collagen *δ*^15^N values above +8‰ analyzed in our study exhibited higher *δ*^15^N_Phe_ values than the others without any overlaps. Most relevant for this study are the canonical trophic position (TP; 1 = primary producers, 2 = primary consumers, 3 = secondary consumers, etc…) estimates based on eqn. 1 (see Methods section) indicating that all cave bears, including those with high *δ*^15^N values for their bulk collagen, had a TP around 2 (TP = 1.8–2.2), similar to published TP-values for cave bears from other sites (Supplementary Table 1).

## Discussion

### Feeding behavior of the cave bears based on AA *δ*^15^N values

**Figure 3:**
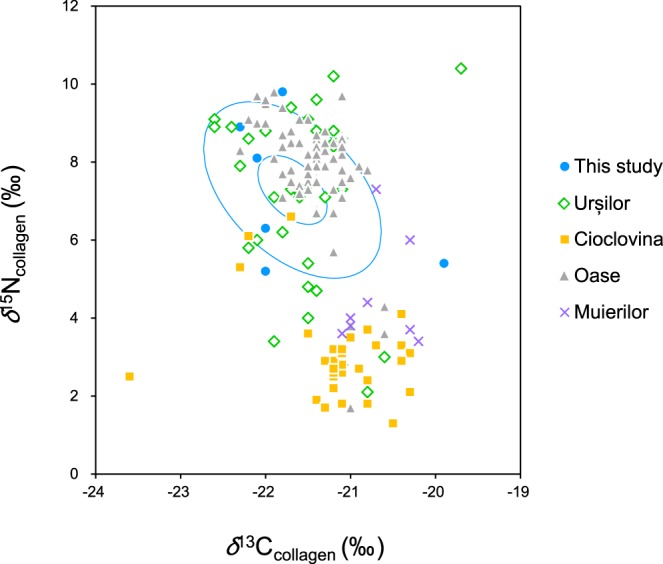
Carbon and nitrogen isotopic composition of bulk collagen for cave bears from the Romanian region. Ellipses indicate 90% and 50% confidence intervals for isotopic compositions of the cave bears analyzed in this study; except for a few individuals like the one with values plotted at the right-upper corner, many cave bears with *δ*^15^N value of collagen around 7–8‰ that might be interpreted as an omnivorous/carnivorous signal can be categorized to highly-plant-dependent feeders.

The bulk collagen *δ*^13^C and *δ*^15^N values are within the range of published data for cave bears from Romanian caves in general (Fig. 3)^24,26^. Thus, the isotopic results that were obtained on single AAs in the present study are likely to apply also to most other cave bears from Romania, including the ones claimed to have been omnivores, carnivores or piscivores, based on the high *δ*^15^N values of their bulk collagen. Our results rule out at least the following possibilities; (i) significant contribution of aquatic resources such as fish to their diets; (ii) carnivory as the main feeding behaviour^54^. The TP estimates for all individuals based on *δ*^15^N of glutamate and phenylalanine were around 2, which rather indicates a highly plant-dependent feeding behavior for all the analyzed cave bears, including those with a high δ^15^N value of their bulk collagen.

The strongest correlation was observed between the *δ*^15^N value of bulk bone collagen and the *δ*^15^N value of phenylalanine that represents *δ*^15^N of the nitrogen source in a food web. This indicates that the bulk collagen *δ*^15^N values of the Romanian cave bears reported here can be explained by the following two possibilities, that are not mutually exclusive: (i) a *δ*^15^N shift of the baseline in this local ecosystem, and/or (ii) the consumption of some specific plants with high *δ*^15^N values by these cave bear individuals. This means that the reported high *δ*^15^N values in the Romanian cave bears’ bulk collagen can be explained by the exclusive consumption of plants rather than by the trophic level effect caused by consumption of animal meat. As shown in Fig. 2, *δ*^15^N_Glx_ and *δ*^15^N_Phe_ values for some individuals, especially the ones with high bulk *δ*^15^N values, overlap with those of strictly herbivorous species, such as late Pleistocene mammoths from other regions, suggesting that a purely herbivorous species could exhibit such carbon and nitrogen isotopic values of bulk collagen in this palaeoenvironmental context^57,58^.

It was shown that there is currently a potential error of ~0.3 units for the TP estimation calculated by the propagation of errors for each parameter in eqn.1^59^. Considering this error together with a general offset of 0.1–0.2 (accuracy) between calculated TP based on eqn.1 and known TP that were estimated based on well-controlled field observations or laboratory feeding experiments for extant animals, our estimates for all individuals are well within the range of acceptable values for herbivorous mammals. In addition to predatory carnivores, fossils that so far yielded TP-values above 2.3 originate from brown bears, wild boars, foxes and badgers^54,60^, in contrast to the cave bears with TPs less than 2.2 analyzed in the present study.

The question remains which kind of plants could have been consumed by the cave bears with bulk collagen high *δ*^15^N values. Mammoth and some Pleistocene fallow deer have been found to exhibit higher *δ*^15^N value and lower *δ*^13^C values relative to the other coeval herbivorous species^43,44^. Some horses from the Late Pleistocene of western Germany also exhibited similar high *δ*^15^N and low *δ*^13^C values and are interpreted as having the same ecological niche as mammoths^61,62^. It is conceivable that the cave bears with high bone collagen *δ*^15^N values fed on plants, since the high values were also found in species with indisputable herbivorous dietary behavior. Possible candidates of such plants with high bulk *δ*^15^N value consumed by cave bears were those consumed by some fallow deer in the Pleistocene^44^, while those consumed by mammoth such as dry, mature grasses and sedges were less likely based on the lower resistance of cave bear teeth against abrasion. Mushrooms, another plant food with high *δ*^15^N values, can be also excluded as an explanation for the high *δ*^15^N values of some cave bears for the two following reasons: (1) mushrooms typically have high *δ*^15^N and high *δ*^13^C values (not low *δ*^13^C values), (2) mushrooms are consumers, like animals, and they display a trophic position value higher than 2^52^, therefore their consumption would lead to a TP significantly higher than 2.

One additional factor specific to bears that has to be considered is hibernation, since unlike the other mammalian taxa discussed above, including *Homo sapiens*, bears hibernate. Indeed, it has been demonstrated that a longer hibernation period caused by climate cooling could result in higher *δ*^15^N of cave bear bulk collagen^63,64^. Although we did not see any statistically significant chronological trend in *δ*^15^N values of AAs, only a limited number of individuals have been analyzed so far with this approach. Further investigations on the possible influence of this factor on the *δ*^15^N values of individual AAs of Ursids should be performed on more extinct cave bears as well as extant bears in order to clarify how nitrogen metabolism such as urea recycling during hibernation possibly affects the *δ*^15^N of AAs in bone collagen^65,66^. Yet, our data currently does not support a major physiological control of the high bulk collagen *δ*^15^N value in bone that remodels intensively during the warm season^30^.

### Evolution of feeding behavior and niche partitioning among cave bear populations

Despite a predominantly herbivorous diet, Romanian cave bears exhibit a very large variation of their isotopic values that probably reflects ecological differences. Such isotopic differences in bone collagen reflect long-term dietary differences, since the turnover of collagen is slow in bone, averaging diet isotopic composition over many years^67^. It is possible that, in contrast to other regions of Europe, the absence of mammoths played a role in allowing cave bears to have access to a more diverse feeding niche and that competition was stronger among cave bears in Late Pleistocene Romania than in other regions, although an exact contemporaneity between the populations with diverse bulk collagen *δ*^15^N values must be examined. If the observed variability in *δ*^15^N values of bulk collagen among the Romanian cave bears was the result of high competition among cave bears in a context of a feeding niche restricted to herbivory, it is possible that some individuals specialized on other plants rather than turned to omnivory including animal food resources. Such a dietary strategy is a very unusual case among bears. Assuming that the herbivory for individual cave bears observed in our study was a general trait across all Late Pleistocene cave bear taxa, the most parsimonious interpretation would be the loss of omnivorous feeding behavior after the divergence of cave bears from the brown/polar bear lineage and before the basal divergence of cave bear lineages (e.g., *U. ingressus* and *U. spelaeus*; see also low bulk *δ*^15^N values for *U. kudarensis*^68^). However, it still remains an open question why this extinct animal that could live over a wide geographical range reaching Transbaikalia in Eastern Siberia with diverse ecological conditions within the mammoth steppe ecosystem finally became extinct^69,70^. It is possible that a dietary adaptation similar to that seen in extant giant panda that consumes large quantities of bamboos but has a macronutrient profile, namely the percent of energy derived from protein, similar to that of carnivores, might have facilitated the full shift for the cave bear lineage towards highly plant-dependent feeding behavior while keeping some ecological plasticity in the mammoth steppe^71^.

Our findings have interesting implications regarding the evolution of herbivory among carnivorans. The foraging strategy of an animal is largely determined by body mass via energetic needs^72^. Cave bear populations were likely adapted to their local habitats, with different altitudes being associated with different features, such as body size, tooth morphology, and tooth wear patterns (e.g., ref. ^21,33,73^). Although the tooth microwear and the isotopic composition of bone collagen reflect the diet of the analyzed individuals at different pre-mortem time periods (e.g., several weeks *vs* several years or more before the death^74^), the observed variation in the bulk collagen *δ*^15^N values for cave bears in the Romanian region possibly reflected a difference in foraging strategy on plants associated with different body sizes^24^. Extant Carnivora species with herbivorous feeding behavior (giant panda, red panda and binturong) have smaller body size and live only in temperate and tropical/subtropical Asia, highlighting the paradox of cave bears that had much larger body sizes and lived in a colder and drier environment. Besides, these three extant species are all on very long evolutionary branches, namely they diverged more than 10 million years ago from their closest relatives. Cave bears, in contrast, share a much more recent ancestor within an omnivorous/carnivorous clade, which is another ecological paradox of this extinct taxon. It is possible that the modern herbivorous carnivorans are relicts of a dietary trend that was much more widespread in the past, and that their rarity is rather due to the recent extinction of the large mammalian species rather than to the impossibility for Carnivora to have a diet restricted to plant food. Our results emphasize the need to investigate more in detail the diversity in food sources, especially plants, consumed by cave bears, as a unique case among Carnivora to further understand the benefits, costs and limitations of herbivory.

## Conclusions

A highly plant-dependent feeding behavior for cave bears was demonstrated by the TP estimates based on *δ*^15^N_Glx_ and *δ*^15^N_Phe_ values. The high *δ*^15^N of bulk bone collagen of some cave bears can be explained by a higher *δ*^15^N baseline of the exploited food chain that is reflected in the *δ*^15^N_Phe_ values, namely plants with high *δ*^15^N values. Therefore, the high *δ*^15^N value of bulk collagen of some cave bears in the Romanian region, a unique feature compared to cave bears from the other regions of Europe analyzed so far, can be explained by the consumption of plants. Further research is needed to clarify which plant species were eaten by those cave bears by using other approaches such as *δ*^13^C analysis of individual AAs to identify protein sources^75,76^. It is also of importance to see if this trend towards herbivory was common for other cave bear populations from broader regions including Asia.

## Methods

### Samples

The Romanian cave sites of Măgura, Cioclovina and Răsuflătoarei, among others, yielded skeletal remains of cave bears^77^. Well-preserved bone specimens of cave bears from these three sites allowed us to examine the feeding behavior of the individuals from the Romanian region. Măgura (46.53169, 22.595969) is a 1,500m-long cave located in the eastern part of the Sighiștel valley part of the Apuseni Natural Park; Cioclovina Uscată (45.576628, 23.134228) is a 2,002m-long cave formed in the Early Cretaceous reef limestones of the Luncani karst platform and situated on the foot of the Southern Carpathians, in the Grădiștea-Muncelului Cioclovina Natural Park; Răsuflătoare cave (45.187800, 21.928000) is located in Caraş-Severin County, Banat Mountains and is a part of the Semenic-Cheile Carașului National Park.

Bone collagen from 6 adult cave bears from the three caves was subjected to *δ*^13^C and *δ*^15^N analysis as well as AA-specific *δ*^15^N analysis. One cave lion collagen sample from the North Sea, an area that was terrestrial during the Late Pleistocene and one horse sample from the site of Lommersum (Germany) from the late Pleistocene, have been analyzed together with the cave bear collagen using the same analytical protocol.

### Collagen extraction and bulk isotopic analysis

The samples were washed in an ultrasonic bath in acetone, rinsed several times with demineralized water, dried at 35 °C for 72 h and crushed to powder of 0.7 mm grain size. Collagen extraction was performed after ref. ^78^ with a minor modification as described in ref. ^79^. In summary, this acid-base-acid method includes a first step of demineralization with 1 M HCl, a second step in 0.125 M NaOH to remove humic acids and lipids, and a final step of gelatinization at pH 2 during 17 h at 100 °C. Isotopic measurements were done using an NC2500 elemental analyzer connected to a Thermo Quest Delta þ XL mass spectrometer. Using the ‘*δ*’ (delta) value, the isotopic compositions are expressed as follows: *δ* (‰) ≡ 10^3^ [*R*_sample_/*R*_standard_ − 1], where the *R* denotes the ^13^C/^12^C ratio for carbon and the ^15^N/^14^N ratio for nitrogen, with the international reference (standards) being V-PDB for *δ*^13^C values and atmospheric nitrogen (AIR) for *δ*^15^N values. Isotopic measurements were normalized to *δ*^13^C values of USGS24 (*δ*^13^C = −16.00‰) and to *δ*^15^N values of IAEA 305 A (*δ*^15^N = +39.80‰). The reproducibility was ±0.1‰ for *δ*^13^C measurements and ±0.2‰ for *δ*^15^N measurements, based on one standard-deviation of the mean of multiple analyses of purified collagen from modern bones and international standards.

### Analytical conditions for *δ*^15^N values of AAs

Bone collagen extracted from the bone samples was hydrolysed with 12 N HCl at 110 °C for 12 h, and derivatized with thionyl chloride/2-propanol (1:4, v/v) at 110 °C for 2 h and subsequently pivaloyl chloride/dichloromethane (1:4, v/v) at 110 °C for 2 h. The *δ*^15^N values of the individual AA derivatives were measured at University of Tübingen using the following systems; A gas chromatography/IRMS (GC/IRMS) using an Agilent Technology 7890B GC coupled to an Elementar Isoprime100 IRMS (Elementar, Germany) via combustion and reduction furnaces. AA derivatives were injected into the GC column (Ultra-2, 50 m × 0.32 mm-i.d. 0.52-µm film thickness; Agilent Technology) in splitless mode at 275 °C. The GC oven temperature was programmed as follows: isothermal hold at 40 °C for 3 min; temperature ramp to 110 °C at 15 °C min^−1^; ramp to 150 °C at 3 °C min^−1^; ramp to 240 °C at 2.5 °C min^−1^; ramp to 270 °C at 6 °C min^−1^; and subsequent holding isothermally at 270 °C for 4 min. Carrier gas (He) flow rate through the GC column was 1.4 ml min^−1^. The CO_2_ generated in the combustion furnace was eliminated by a liquid nitrogen trap. Standard mixtures of ten AAs with known *δ*^15^N values were injected into the GC/IRMS every two to five runs to confirm reproducibility of the isotope measurements. The precision of the reference mixtures was 0.65–0.90‰. Nitrogen isotopic composition of maximally the following eight AAs was determined as their N_2_ gas peaks from derivatives showed fine resolution on the GC/IRMS chromatogram: valine (Val), leucine (Leu), isoleucine (Ile), proline (Pro), serine (Ser), glutamate (Glx), phenylalanine (Phe) and hydroxyproline (Hyp). All reported *δ*^15^N values for glutamate included the contributions from the α-amino group of glutamic acid and glutamine, as glutamine is converted to glutamic acid during acid hydrolysis. Data for alanine and glycine were not obtained for all samples due to relatively large peak sizes in this study.

Using the equation below based on *δ*^15^N of glutamate and phenylalanine, we estimated the trophic position of cave bears in terrestrial C_3_-plant-based ecosystem: TP = [(*δ*^15^N_Glx_ − *δ*^15^N_Phe_ − *β*)/TDF] + 1 (eqn. 1), where *δ*^15^N_Glx_, *δ*^15^N_Phe_, *β* and TDF indicate *δ*^15^N of glutamate, *δ*^15^N of phenylalanine and the isotopic offset between glutamate and phenylalanine (*δ*^15^N_Glx_ − *δ*^15^N_Phe_ value) in primary producers, the trophic discrimination factor, respectively^60^. The values in the equation are based on the fact that some “source” AAs show little isotopic change through each trophic step (e.g. 0.4 ± 0.4‰ for phenylalanine) whereas other “trophic” AAs fractionate with trophic steps (e.g. 8.0 ± 1.1‰ for glutamate)^47–49^. We adopted 8.4‰ for the *β* value for terrestrial C_3_ vascular plants^49,80^, and 7.2‰ for the TDF value^81^, in this study.

### Radiocarbon dating

Cave bear collagen was dated directly using AMS radiocarbon dating. Radiocarbon dating was performed by the Labor für Ionenstrahlphysik at Eidgenössische Technische Hochschule—ETH in Zurich (Switzerland). The obtained radiocarbon ages were calibrated using the OxCal v4.2.4 software using the IntCal13 atmospheric curve^82,83^. All dates were calibrated to BP dates with 2σ (95.4%) probability.

## Inferring molecular ages

### Ancient DNA extraction and library building

For two samples lacking radiocarbon dates (USR10 and USR67), molecular tip-dating was performed. DNA was extracted from 50 mg of cave bear petrous bones following the protocol of ref. ^84^, with reduced centrifugation speeds as described in ref. ^85^. Prior to library building, each DNA extract has been quantified with the Qubit 2.0 fluorometer with high sensitivity reagents (Thermo Fisher Scientific) by using one microlitre out of 25 μL extract. To support the validity of the measurements, positive and negative controls were also measured. Illumina single-stranded libraries were then prepared following the protocol of ref. ^86^, after the removal of uracil residues and abasic sites. The optimal number of library amplification PCR cycles has been assessed with qPCR as described in ref. ^85^. A volume of 80 μL including 20 μL template library, Accuprime Pfx DNA polymerase and eight base-pair tailed-primers was used for Indexing PCR to generate dual-indexed library molecules. Amplified libraries were purified using commercial silica spin-columns (Qiagen MinElute). Prior to single-end sequencing on Illumina platforms, library concentration and length distribution were quantified using Qubit 2.0 and 2200 TapeStation (Agilent Technologies), respectively.

### Sequencing and bioinformatic analyses

Shotgun sequencing was performed on an Illumina NextSeq 500 sequencing platform. We obtained 75 bp single-end reads using the NextSeq 500/550 High Output Kit v2 (75 cycles) sequencing kit, following the procedures described in ref. ^87^. Raw reads were trimmed with minimum overlap of one nucleotide and reads shorter than 30 bp were discarded using cutadapt v1.12^88^. The processed reads were mapped to the reference mitogenome sequence of *Ursus spelaeus* (Genbank Acc. No. EU327344, ref. ^89^) with default parameters using the bwa v0.7.15 “aln” algorithm^90^. The alignment was then filtered for mapping quality (Q > 30), sorted by read position and potential PCR duplicates were removed using SAMtools v0.1.19^90^.

### Mitochondrial genome reconstruction and alignment

ANGSDv0.920 was used to generate consensus sequences for both individuals^91^. Using the option -doFasta 3 that takes into account bases with highest effective depth^92^, the mitochondrial consensus has been constructed only considering read mapping and Phred base quality scores >30. The two mitogenomes were aligned to a published alignment of cave bear mitochondrial sequences^15^. The repetitive section of the d-loop was removed from the alignment because it could not be aligned unambiguously. The final alignment comprised 19 sequences of 16,468 nucleotides, publicly available (Dryad database and GenBank). The GenBank accession numbers of the mitogenomes generated in this study are: MN311249 for USR10 and MN311250 for USR67.

### Molecular tip-dating

Ages of the two samples were estimated using Bayesian phylogenetic tip dating^93^, a method shown to provide accurate age estimates for Late Pleistocene European cave bears^15^. PartitionFinder v.2.1.1 has been used for data partitioning and substitution model selection^94^. All substitution models available in BEAST v.1.8.2 were considered and the best partitioning scheme has been computed under the Bayesian Information Criterion using linked branch lengths and the greedy search algorithm (Supplementary Table 2)^95^.

Tip-dating was performed separately for each sample by analyzing its mitochondrial sequence in BEAST v1.8.2, using median ^14^C dates as calibration from the dataset in ref. ^15^ (Supplementary Tables 3). For each tip date its posterior distribution has been sampled using a uniform, uninformative prior of zero to one million yBP. To accommodate changes in effective population size during the time-span of the tree, a piecewise-constant coalescent Bayesian Skyline population model was selected. Unlinked strict molecular clocks were set for each data partition with the posterior distribution of the substitution rate of each sample with a uniform, uninformative prior of zero to 20% mutations per million years. The software Tracer v1.6 was used to assess if the MCMC chain had run for a sufficient length to achieve burn-in and adequate sampling of all parameters (ESS > 200)^96^. The Maximum Clade Credibility Tree was selected from the posterior sample using TreeAnnotator and visualised in FigTree (Supplementary Fig. 1).

## Supplementary information

Supplementary information.
