# Author Correction: Heavy reliance on plants for Romanian cave bears evidenced by amino acid nitrogen isotope analysis

**DOI:** 10.1038/s41598-020-75177-4

**Published:** 2020-10-28

**Authors:** Yuichi I. Naito, Ioana N. Meleg, Marius Robu, Marius Vlaicu, Dorothée G. Drucker, Christoph Wißing, Michael Hofreiter, Axel Barlow, Hervé Bocherens

**Affiliations:** 1 Department of Geosciences, Biogeology, University of Tübingen, Hölderlinstraße 12, 72074 Tübingen, Germany; 2 Nagoya University Museum, Nagoya University, Furo-cho, Chikusa-ku, Nagoya, 464-8601 Japan; 3 “Emil Racoviţă” Institute of Speleology, Romanian Academy, Calea 13 Septembrie, nr. 13, Sector 5, 050711 Bucharest, Romania; 4 Senckenberg Centre for Human Evolution and Palaeoenvironment (S-HEP), University of Tübingen, Hölderlinstraße 12, 72074 Tübingen, Germany; 5 Institute for Biochemistry and Biology, Faculty for Mathematics and Natural Sciences, University of Potsdam, Karl-Liebknecht-Str. 24-25, 14476 Potsdam, OT Golm Germany; 6 School of Science and Technology, Nottingham Trent University, Clifton Lane, Nottingham, NG11 8NS UK

Correction to: *Scientific Reports* 10.1038/s41598-020-62990-0, published online 20 April 2020

This Article contains errors in Table 1 where two column headings are incorrect.

“*d*^13^C (‰)” and “*d*^15^N (‰)”.

should read:

“*δ*^13^C (‰)” and “*δ*^15^N (‰)”, respectively.

