# Supplementary material for: Heavy reliance on plants for Romanian cave bears evidenced by amino acid nitrogen isotope analysis
Source: Sci Rep. 2020 Apr 20;10:6612. doi: 10.1038/s41598-020-62990-0 (PMC7170912; doi:10.1038/s41598-020-62990-0)
Supplement: Supplementary file 1 — Supplementary information. [file 41598_2020_62990_MOESM1_ESM.docx]

Heavy reliance on plants for Romanian cave bears evidenced by amino acid nitrogen isotope analysis

**Supplementary Information**

*Authors*: Yuichi I. Naito^1,2,*^, Ioana N. Meleg^3,*^, Marius Robu^3^, Marius Vlaicu^3^, Dorothée G. Drucker^4^, Christoph Wißing^1^, Michael Hofreiter^5^, Axel Barlow^5^, and Hervé Bocherens^1,4^

*Affiliations*: ^1^Department of Geosciences, Biogeology, University of Tübingen, Hölderlinstraße 12, 72074 Tübingen, Germany

^2^Nagoya University Museum, Nagoya University, Furo-cho, Chikusa-ku, Nagoya 464-8601, Japan

^3^"Emil Racoviță" Institute of Speleology, Romanian Academy, Calea 13 Septembrie, nr. 13, 050711, Sector 5, Bucharest, Romania

^4^Senckenberg Centre for Human Evolution and Paleoenvironment (S-HEP), University of Tübingen, Hölderlinstraße 12, 72074 Tübingen, Germany

^5^Institute for Biochemistry and Biology, Faculty for Mathematics and Natural Sciences
Karl-Liebknecht-Str. 24-25, 14476 Potsdam OT Golm


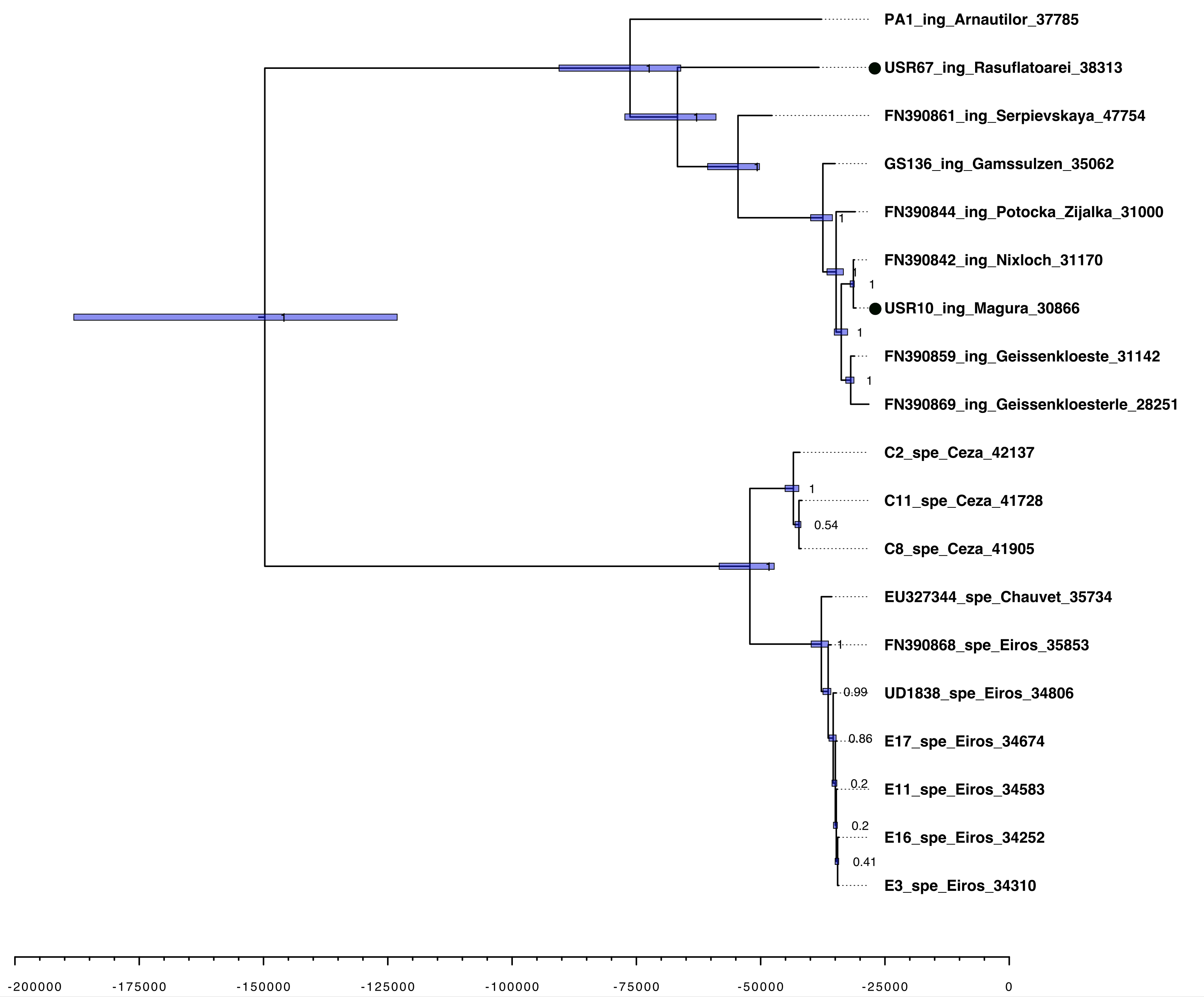


**Supplementary Figure 1. Time-calibrated phylogeny of the cave bears.** The lower scale shows years BP. Branch labels indicate posterior clade probabilities. Nodes are centred on the median estimated divergence time, and bars show the 95% HPD. Black circles: estimated age of the two Romanian samples generated in this study, indicated at the end of the sample. The Maximum Clade Credibility Tree from the BEAST analysis based on complete mitogenomes includes representatives of the two main cave bear clades: *ingressus* and *spelaeus.* The young Romanian individual (USR10) is nested within a clade comprising other European cave bears, while the middle-aged Romanian individual (USR67) forms a distinct clade.

**Supplementary Table 1. Nitrogen isotopic compositions** **(**‰ ***vs* AIR) of individual amino acids and trophic position estimates.**

| Lab. ID | Species name | Val | Leu | Ile | Pro | Ser | Glx | Phe | Hyp | TP (C_3_) |
| --- | --- | --- | --- | --- | --- | --- | --- | --- | --- | --- |
|  |  |  |  |  |  |  |  |  |  |  |
| USR10 | - | 10.6 | 10.4 | 8.4 | 11.7 | 2.3 | 12.1 | 12.4 | 10.4 | 2.1 |
| USR11 | - | N.R. | 11.8 | 12.0 | 15.9 | N.R. | 14.0 | 14.0 | 13.1 | 2.2 |
| USR22 | - | 10.4 | 8.7 | 6.4 | 13.5 | 1.0 | 10.4 | 10.6 | 10.1 | 2.1 |
| USR25 | - | 12.7 | 10.9 | 7.6 | 14.0 | 2.4 | 11.9 | 14.8 | 12.6 | 1.8 |
| USR65 | - | N.R. | N.R. | N.R. | 12.3 | N.R. | 13.5 | 15.2 | 12.1 | 1.9 |
| USR67 | - | 9.5 | 6.8 | 0.9 | 10.6 | -2.3 | 10.0 | 9.6 | 8.4 | 2.2 |
|  |  |  |  |  |  |  |  |  |  |  |
| *Reference fauna* | |  |  |  |  |  |  |  |  |  |
| LOM 16 | *Equus ferus* | 5.1 | 5.7 | 4.6 | 10.9 | N.R. | 6.1 | 7.6 | 8.4 | 2.0 |
| Leo 01 | *Panthera spelaea* | 15.0 | 8.4 | N.R. | 13.7 | 0.4 | 12.7 | 8.2 | 12.2 | 2.8 |
|  |  |  |  |  |  |  |  |  |  |  |

N.R. not reported due to small peak size or overlap with unknown peaks

**Supplementary Table 2. Best partitioning scheme selected by PartitionFinder for Bayesian tip dating and Maximum Clade Credibility Tree.**

| Partition | Best Model | MtDNA elements |
| --- | --- | --- |
| 1 | HKY+X | Leu2, Lys, Ile, His, Asp, Arg, Gly, Asn, Val, Ser1, 16s_pt2, Glu, Phe, Trp, Ser2, Ala, ND4_CP1, ND5_CP1, ATP8_ATP6_CP3, ND2_CP1, ND6_CP2, 12s, 16s_p1 |
| 2 | K80 | ND3_CP1, COX3_CP1, Cys, CYTB_CP1, Leu1, COX1_CP1, ND1_CP1, Met, COX2_CP1, ND4L_CP1 |
| 3 | HKY+X | COX2_CP2, ND4L_CP2, ND5_CP2, ND3_CP2, ATP8_ATP6_CP1, ND2_CP2, ND1_CP2, COX1_CP2, ND4_CP2, CYTB_CP2, COX3_CP2 |
| 4 | HKY+X+G | ND6_CP1, ND1_CP3, ND4_CP3, CYTB_CP3, ND2_CP3, ND5_CP3 |
| 5 | HKY+X | Gln, Pro, COX2_CP3, ND6_CP3, ND3_CP3, COX3_CP3, ND4L_CP3, Tyr, Thr, D-loop, ATP8_ATP6_CP2, COX1_CP3 |

**Supplementary Table 3. Details of ^14^C dated mitochondrial sequences used for Bayesian tip dating and Maximum Clade Credibility Tree.**

| Sample ID | Taxon | Locality | Uncal. Age yBP | Uncertainty ± | Cal age yBP* | Uncertainty ± | Reference ^14^C |
| --- | --- | --- | --- | --- | --- | --- | --- |
| GS136 | *ingressus* | Gamssulzen Austria | 31026 | 500 | 35062 | 966 | Fortes et al. 2016 |
| FN390869 | *ingressus* | Geissenkloesterle Germany | 24210 | 100 | 28251 | 310 | Stiller et al. 2010 |
| FN390859 | *ingressus* | Geissenkloesterle Germany | 27180 | 150 | 31142 | 215 | Stiller et al. 2010 |
| FN390842 | *ingressus* | Nixloch Austria | 27230 | 140 | 31170 | 208 | Hofreiter et al 2002 |
| FN390844 | *ingressus* | Potocka Zijalka Slovenia | 26900 | 110 | 31000 | 190 | Hofreiter et al 2002 |
| PA1 | *ingressus* | Romania | 33600 | 400 | 37785 | 1083 | Fortes et al. 2016 |
| FN390861 | *ingressus* | Serpievskaya Russia | 44050 | - | 47754 | 576 | Stiller et al. 2010 |
| C11 | *spelaeus* | A Ceza Spain | 37240 | 330 | 41728 | 516 | Fortes et al. 2016 |
| C8 | *spelaeus* | A Ceza Spain | 37490 | 370 | 41905 | 547 | Fortes et al. 2016 |
| C2 | *spelaeus* | A Ceza Spain | 37830 | 390 | 42137 | 548 | Fortes et al. 2016 |
| EU327344 | *spelaeus* | Chauvet France | 31870 | 300 | 35734 | 643 | Bon et al 2008 |
| E3 | *spelaeus* | Eiros Spain | 30150 | 606 | 34310 | 1186 | Pérez Rama et al 2011 |
| E16 | *spelaeus* | Eiros Spain | 30210 | 160 | 34252 | 339 | Fortes et al. 2016 |
| E11 | *spelaeus* | Eiros Spain | 30660 | 190 | 34583 | 389 | Fortes et al. 2016 |
| E-VD-1838 | *spelaeus* | Eiros Spain | 30737 | 500 | 34806 | 931 | Fortes et al. 2016 |
| E17 | *spelaeus* | Eiros Spain | 30760 | 170 | 34674 | 365 | Fortes et al. 2016 |
| FN390868 | *spelaeus* | Eiros Spain | 31390 | 865 | 35853 | 1961 | Pérez Rama et al 2011 |

- ^14^C dates were 2-sigma calibrated using 2-sigma calibrated using OxCal v4.3.2 (Bronk Ramsey 2017), based on the IntCal13 atmospheric curve (Reimer et al. 2013)

**References**

Fortes, G. G. et al. Ancient dna reveals differences in behaviour and sociality between brown bears and extinct cave bears. *Molec. Ecol.* **25**, 4907–4918 (2016).

Stiller, M. et al. Withering away - 25,000 years of genetic decline preceded cave bear extinction. *Mol. Biol. Evol.* **27**, 975-978 (2010).

Hofreiter, M. et al. Ancient DNA analyses reveal high mitochondrial DNA sequence diversity and parallel morphological evolution of late pleistocene cave bears. *Mol. Biol. Evol.* **19**, 1244–1250 (2002).

C. Bon, N. et al. Deciphering the complete mitochondrial genome and phylogeny of the extinct cave bear in the Paleolithic painted cave of Chauvet. *Proc. Natl. Acad. Sci. USA* **105**, 17447–17452 (2008).

M. Pérez-Rama et al. Effects of hibernation on the stable isotope signatures of adult and neonate cave bears. *Quaternaire Hors série* **4**, 79–88 (2011).

C. Bronk Ramsey. Methods for Summarizing Radiocarbon Datasets. *Radiocarbon* **59**, 1809-1833 (2017).

Reimer, P. J. et al. IntCal13 and Marine13 Radiocarbon Age Calibration Curves 0–50,000 Years cal BP. *Radiocarbon* **55**, 1869-1887 (2013).
